# Cancer Therapy: Shooting for the Moon

**DOI:** 10.1002/cpt.655

**Published:** 2017-04-18

**Authors:** JSH Lee, GM Blumenthal, RJ Hohl, S‐M Huang

**Affiliations:** ^1^ Office of the Director, National Cancer Institute National Institutes of Health Bethesda Maryland USA; ^2^ Office of Hematology & Oncology Products, Center for Drug Evaluation and Research Food and Drug Administration Silver Spring Maryland USA; ^3^ Penn State Cancer Institute Pennsylvania State University Hershey Pennsylvania USA; ^4^ Office of Clinical Pharmacology, Office of Translational Sciences, Center for Drug Evaluation and Research Food and Drug Administration Silver Spring Maryland USA

The drug development paradigm is rapidly evolving from its longstanding “siloed” corporate structure to one based more on partnerships among industry, academia, and the regulatory agencies. These partnerships take advantage of the individual strengths that have existed in each sector but reflect the need for enhanced collaboration. In 2016, the White House Cancer Moonshot brought this need for increased collaboration in oncology to the public's attention through recognition that in this era of advanced technologies and rapid accumulation of data, there is not only a need to have knowledge readily accessible and interactive, but also a need for strategic partnerships among public and private sectors to allow key *in silico,* basic, clinical, and population‐based experiments to be conducted that will advance the field. To help define a national cancer research blueprint, the National Cancer Advisory Board assembled a Blue Ribbon Panel of 28 scientific experts and other stakeholders that provided transformative recommendations to accelerate progress against cancer after consulting more than 150 experts and reviewing more than 1,600 suggestions from the public.^1^ With strong bipartisan support, the 21st Century Cures bill was passed by Congress on December 7, 2016, with funding “to support cancer research, such as the development of cancer vaccines, the development of more sensitive diagnostic tests for cancer, immunotherapy, and the development of combination therapies, and research that has the potential to transform the scientific field, that has inherently higher risk, and that seeks to address major challenges related to cancer.”^2^ These new resources will help not only to implement recommendations of the Blue Ribbon Panel, but also to encourage continued cooperation amongst academia, government, and industry to develop new partnerships that minimize the potential of generating additional siloes in the future.

In this issue, devoted to the implications and advances in oncology therapeutics, novel descriptions of new paradigms for many components of the drug development process are presented. If advanced appropriately, these partnerships have the potential to substantially change the way the drug development paradigm is approached. The implications of success will not only enhance the availability of better oncology therapies available to US patients, but also make these therapies available to the global community. These are important approaches that will need to be carefully assessed in the coming years, but for the moment serve as a new baseline and present an exciting glimpse into the future. It will be important for organizations, such as the American Society for Clinical Pharmacology & Therapeutics and its membership, to take the lead in judiciously and thoughtfully shaping and evaluating these approaches.

## EMERGING DRUG DEVELOPMENT PARADIGM AND REGULATORY REVIEW AND APPROVAL PROCESS

Oncology drug development has seen tremendous progress in recent years. With the remarkable growth in the understanding of the molecular basis of cancer etiology and the development of novel therapeutic targets, innovative approaches in drug development and regulatory approval pathways, the US Food and Drug Administration (FDA) has granted significant approvals of new targeted therapies and immunotherapies (**Tables** **1**, **2**),^3^, ^4^ in addition to biosimilar products.^5^, ^6^

**Table 1:** Selected oncology targeted and immunotherapy approvals since 2013 (adapted from Blumenthal *et al* 3)

Indication	Therapy (target)
Anaplastic LCL	Brentuximab vedotin (CD30)
ALL (Philadelphia negative)	Blinatumomab (CD19/CD3)
B‐cell NHL	Idelalisib (PI3K delta)
Basal cell	Sonidegib (hedgehog pathway)
Breast cancer	Palbociclib (CDK 4 and 6); pertuzumab (HER2/neu)
CLL	Ibrutinib (BTK); idelalisib (PI3K delta); obinutuzumab, ofatumumab (CD20); venetoclax (BCL‐2/17p deletion)
Follicular lymphoma	Obinutuzumab (CD20)
HNSCC	Nivolumab, pembrolizumab (PD‐1)
Hodgkin's lymphoma	Brentuximab vedotin (CD30); nivolumab (PD‐1)
Mantle cell lymphoma, WM	Ibrutinib (BTK)
Metastatic melanoma	Cobimetinib, trametinib (BRAF/MEK); vemurafenib, dabrafenib (BRAF/MEK); Ipilimumab (CTLA4); nivolumab, pembrolizumab (PD‐1)
Metastatic NSCLC	Afatinib, erlotinib, gefitinib, osimertinib (EGFR); alectinib, ceritinib, crizotinib (ALK); crizotinib (ROS‐1); atezolizumab, pembrolizumab, nivolumab (PD‐1/PD‐L1)
Multiple myeloma	Carfilzomib, Ixazomib (proteasome); elotuzumab (SLAMF7)
Ovarian	Olaparib, rucaparib (PARP/ BRCA)
Renal cell carcinoma	Nivolumab (PD‐1)
Soft tissue sarcoma	Olaratumab (PDGFR‐alpha)
Urothelial	Atezolizumab (PD‐L1)

Anaplastic LCL, Anaplastic large cell lymphoma; ALL, acute lymphoblastic leukemia; CLL, chronic lymphocytic leukemia; HNSCC, head and neck squamous cell carcinoma; metastatic NSCLC, metastatic non‐small cell lung cancer; WM, Waldenström's macroglobulinemia. Ref: http://www.accessdata.fda.gov/scripts/cder/daf/

**Table 2:** FDA‐approved drugs with companion diagnostics^a^ (updated from Pacanowski and Huang 2016^4^)

Drug generic name (trade name)	Biomarker and disease^a^	Device trade name(s)
Afatinib (Gilotrif); Gefitinib (Iressa)	EGFR mutations in non‐small cell lung cancer	therascreen EGFR RGQ PCR Kit
Erlotinib (Tarceva); Osimertinib (Tagrisso)	EGFR mutations in non‐small cell lung cancer	cobas EGFR Mutation Test V2 (for both tissue and plasma)
Pembrolizumab (Keytruda)	PD‐L1 expression in non‐small cell lung cancer	PD‐L1 IHC 22C3 pharmDx
Crizotinib (Xalkori)	ALK rearrangements in non‐small cell lung cancer	VYSIS ALK Break Apart FISH Probe Kit, VENTANA ALK (D5F3) CDx Assay
Tramatenib (Mekinist); Dabrafenib (Tafinlar)	BRAF mutations in melanoma	THxID BRAF Kit
Vemurafenib (Zelboraf); Cobimetinib (Cotellic) with Vemurafenib	BRAF mutations in melanoma	COBAS 4800 BRAF V600 Mutation Test
Trastuzumab (Herceptin)	HER2 expression in breast cancer	INFORM HER‐2/NEU, PATHVYSION HER‐2 DNA Probe Kit, PATHWAY ANTI‐HER‐2/NEU (4B5) Rabbit Monoclonal Primary Antibody, INSITE HER‐2/NEU KIT, SPOT‐LIGHT HER2 CISH Kit, Bond Oracle Her2 IHC System, HER2 CISH PharmDx Kit, INFORM HER2 DUAL ISH DNA Probe Cocktail
Trastuzumab (Herceptin); Pertuzumab (Perjeta); Ado‐trastuzumab emtansine (Kadcyla)	HER2 expression in breast cancer and gastric cancer^b^	HER2 FISH PharmDx Kit, HERCEPTEST
Olaparib (Lynparza)	BRCA variants in ovarian cancer	BRACAnalysis CDx
Rucaparib (Rubraca)	BRCA alterations in ovarian cancer	FoundationFocus CDx*BRCA* Assay‐ next generation sequencing
Cetuximab (Erbitux); Panitumumab (Vectibix)	EGFR expression in colorectal cancer	DAKO EGFR PharmDx Kit,
	KRAS mutations in colorectal cancer	therascreen KRAS RGQ PCR Kit, cobas KRAS Mutation Test
Imatinib mesylate (Gleevec)	c‐kit expression in gastrointestinal stromal tumors	DAKO C‐KIT PharmDx
	KIT D816V Mutation Aggressive systemic mastocytosis	*KIT* D816V Mutation Detection by PCR
	PDGFRB gene rearrangement myelodysplastic syndrome/ myeloproliferative disease	PDGFRB FISH
Deferasirox (Exjade)	Liver iron concentrations in non‐transfusion dependent thalassemia	Ferriscan
Venetoclax (Venclexta)	17p deletion in chronic lymphocytic leukemia	Vysis CLL FISH PROBE KIT

a Includes only indications for which an FDA‐cleared or ‐approved companion diagnostic is available.

b Gastric cancer indication is for trastuzumab only.

Adapted from the FDA's List of Cleared or Approved Companion Diagnostic Devices (In Vitro and Imaging Tools), http://www.fda.gov/MedicalDevices/ProductsandMedicalProcedures/InVitroDiagnostics/ucm301431.htm.

For example, with the marked clinical efficacy of crizotinib for non‐small cell lung cancer (NSCLC), various second‐generation anaplastic lymphoma kinase (ALK) inhibitors have been or are being developed, mostly for use in crizotinib‐resistant settings. Central nervous system (CNS) antitumor activity is critical for specific ALK inhibitors to be used for first‐line treatment to reduce or delay the rate of brain metastasis in patients with ALK‐positive NSCLC.^7^ Currently, many bispecific antibodies are being developed for cancer immunotherapy. Yuraszeck *et al*.^8^ described unique challenges and experiences in the preclinical and the clinical development of blinatumomab, a bispecific T‐cell engaging antibody, pointing out the critical need to optimize dose and schedule of combination therapy and the potential utility of quantitative systems pharmacology and other pharmacometric tools (e.g., physiologically based pharmacokinetic modeling). Another area of active development is cancer prevention. Today, vaccination is available to prevent some cancers, for example, human papillomavirus (HPV) vaccine for the prevention of cervical, vulvar, vaginal, and anal cancers. The strong evidence from the literature also shows that vaccination with hepatitis B vaccine can reduce the incidence of hepatocellular carcinoma. However, development of agents to prevent cancer whose causes are not associated with infection, although promising, is more challenging and still at an early stage.^9^

The FDA has provided various regulatory pathways to expedite the development and approval of drugs. For 2016, 73% of the approvals of novel drugs used one of the expedited pathways, and of the six oncology drugs approved for therapeutic or diagnostic purposes (atezolizumab, flucidovine‐18, gallium Ga 68 dotatate, olaratumab, rucaparib, and venetoclax), all were designated Priority Review, four Breakthrough Therapy Designation, four accelerated approval, and one Fast Track designation.^10^ As pointed out by Blumenthal *et al*.,^3^ the FDA has approved several breakthrough‐designated drugs based on expansion cohorts in phase I clinical trials. In addition to this novel “seamless drug development” paradigm,^11^ the increasing use of master protocols that are genomically driven, including basket trials (such as the NCI‐MATCH trial^12^) and umbrella trials (such as Lung‐Map^13^), is promoting important collaborations among stakeholders for efficient development of oncology drugs. While the traditional time‐to‐event endpoints (progression‐free survival (PFS) and overall survival) are generally used for regular approval, objective response rate (ORR) and duration of response (DoR) can often be used for accelerated approval, especially in single‐arm trials.^3^ However, these latter data (ORR and DoR) that may be used for drug approvals may not be suitable for health economic appraisal.^14^ In addition, the use of PFS as evaluated via RECIST (Response Evaluation Criteria in Solid Tumors) may not be appropriate for locally administered oncolytic viral therapies.^15^

The use of model‐informed drug development (MIDD) strategies has increased in the past few years. PDUFA VI (for fiscal years 2018–2022) included a commitment to “facilitate the development and application of exposure‐based, biological, and statistical models to derive from preclinical and clinical data sources.”^16^ To optimize individual dosing regimens, it is critical to have appropriate clinical pharmacology studies, including timely food effect evaluation,^17^ dose‐ranging trial designs, and best practices in pharmacometric methodologies.^18^ Despite the increasing use of MIDD in drug development and regulatory review, the use of models to inform dosing in specific subpatient groups to improve treatment outcomes appears to be limited and requires wider interdisciplinary collaborations.^19^

## ACCELERATING THE PROGRESS AGAINST CANCER: NATIONAL INFRASTRUCTURE PILOTS AND PARTNERSHIPS

An average cancer patient may be seen by more than a dozen different providers during his/her individual patient journey. The ability to aggregate a patient's data is currently difficult, if not impossible. A major recommendation from policy and research experts is to enable the creation of a learning healthcare system for cancer that will allow us to glean knowledge and experience from every cancer patient.^20^, ^21^ Only then will we be able to use data to enhance, improve, and inform the journey of every cancer patient from the point of diagnosis through survivorship. This can be thought of analogously to Waze, a community‐based traffic and navigation app in which millions of drivers work together daily toward a common goal of finding the best route to their destinations. Drivers that take local roads are able to share their experiences with similar drivers, while at the same time providing useful information to high‐speed drivers that choose to take highways. While both may arrive at the same destination, their shared individual experiences will help inform the next driver who must determine the best path to take on a similar journey. To achieve a “Waze for cancer,” we must go beyond the traditional notion of simply aggregating data and move towards a structure that allows for reuse of harmonized data, as demonstrated by the National Cancer Institute's (NCI's) Genomic Data Commons and NCI Genomic Cloud Pilots.^20^ Orthogonal datasets from the same patient that will allow machine learning algorithms to challenge existing dogmas will also be needed. The partnership among the Department of Veterans Affairs, Department of Defense, and NCI will challenge the status quo by examining a patient's genes (genomic analysis), the expression of these genes in the form of proteins (proteomic analysis), as well as medical images associated with the same patient to create the nation's first system in which cancer patients are routinely screened for genomic abnormalities, proteomic information, and imaging to match their tumor types to targeted therapies. The Applied Proteogenomics OrganizationaL Learning and Outcomes (APOLLO) network will generate molecular datasets from thousands of patients, so that when clinical annotation is layered on top, the network will help to predict which patients will respond to which therapies.^22^ This analysis could either inform new combinations or use existing novel therapies within the newly established NCI formulary^23^ to better understand primary resistance and secondary resistance in patients. To provide temporal molecular characterization of patients for machine‐learning purposes, subsets of data being harmonized and generated by a public–private partnership, Blood Profiling Atlas in Cancer (BloodPAC), could be used.^24^ BloodPAC brings together 25 state‐of‐the‐art biotech companies, pharmaceutical partners, and academic labs focused on developing assays to analyze blood. The collaboration has already shared raw data from over a dozen studies and will work with the APOLLO network through the Department of Defense. Finally, data elements describing immediate patient response from APOLLO and BloodPAC will be developed and defined in a uniform manner with input from Centers for Medicare/Medicaid (CMS) and FDA, enabling multidimensional analysis of these datasets by other payers to assess whether similar approaches will improve health outcomes for their relevant populations, as described broadly by the CMS Oncology Care Model.^25^ The examples given above, and included in this issue, represent only a snapshot of infrastructures and partnerships that will be needed. In 2017, continued implementation of these efforts will lay the foundation critical to achieving the ambitious recommendations from the NCI Blue Ribbon Panel with additional funding opportunities updated on a routine basis.^26^

## CONCLUSION AND FUTURE DIRECTIONS

Also as part of the Cancer Moonshot program, on June 29, 2016, the FDA was instructed to leverage the skills of regulatory scientists and reviewers across the various product centers (drugs, biologics, and devices) to create the Oncology Center of Excellence (OCE). The goal of the OCE is to further expedite the development of new combination products and support an integrated approach to addressing cancer as a disease.^27^ On January 19, 2017, the agency formally announced the establishment of the OCE and appointed Dr. Richard Pazdur as OCE director.^28^

The OCE will seek to emulate both academic and cancer care centers, which are increasingly organized in multidisciplinary models to enhance collaboration that is critical when confronting a complex and rapidly evolving disease such as cancer.^29^ The OCE will continue to incorporate the patient view into regulatory decision‐making and will support innovation to better integrate the multiple disease and diagnostic options to further patient care.^29^

## DISCLAIMER

The views expressed in this article are those of the authors and do not necessarily reflect the official views of the FDA or NCI.

## CONFLICT OF INTEREST

The authors declare no conflicts of interest.
